# An Unusual Cause of Recurrent Chylothorax: Gorham Syndrome

**Published:** 2014-05-21

**Authors:** Rahşan Özcan, Ahmet Alptekin, Şenol Emre, Sebuh Kuruoğlu, Muharrem Inan, Gonca Topuzlu Tekant

**Affiliations:** 1 Department of Pediatric Surgery, Istanbul University, Cerrahpaşa Medical Faculty, Istanbul, Turkey;; 2 Department of Radiology, Istanbul University, Cerrahpaşa Medical Faculty, Istanbul, Turkey;; 3 Department of Orthopedic Surgery, Istanbul University, Cerrahpaşa Medical Faculty, Istanbul, Turkey;

**Dear Sir,**

Gorham syndrome, which is also called diffuse lymphangiomatosis, is characterised by excessive osteolysis due to uncontrolled lymphatic or vascular proliferation involving bones, of unknown etiology. Chylothorax may accompany Gorham syndrome in case of invasion of lymphatic vessels, or thoracic duct due to neighboring affected ribs, scapula or vertebrae. Chylothorax and vertebral invasion is seen in 17-20% of patients. Even though there are many different suggested treatment modalities; Gorham syndrome with chylothorax has a very high morbidity and mortality.[1-3] This case report presents a patient with Gorham syndrome and chylothorax who was treated successfully with interferon alpha-2b.

A 7-year-old child with a history of coughing, fever and dyspnea for 15 days was admitted to another clinic. Due to bilateral chylous pleural effusion bilateral tube thoracostomies were performed. No underying cause could be found. He was treated with total parenteral nutrition (TPN) and after chest tube removal, discharged with a MCT (middle chain triglycerids) diet. Two months after discharge, he was admitted to our clinic with dyspnea and coughing. On chest x-ray, bilateral pleural effusion was founded of which right side was worse. Thorax MRI demonstrated bilateral pleural effusion and patchy type osteolytic changes of the thoracic vertebra (Fig. 1). He was fed with MCT rich diet and intravenous somatostatin treatment used with a dose of 24 microgram/kg/day for 21 days. At 15th day of treatment, steroid was added at a dose of 2 mg/kg/day and tapered over 3 months. After 3 months, recurrence of the pleural effusion was noted and following thoracentesis interferon alpha 2b treatment was started. Interferon alpha-2b was given 3 days in a week at a dose of 1500000 units/m2/day in first two weeks and for the following 9 months 3 days in a week with a dose of 3000000 units/m2/day. No side effect of interferon was observed. Chest x-ray at 3rd month of treatment demonstrated minimal pleural effusion in the right hemithorax. On follow-up, the patient has been symptom free for two years and is not receiving any medication or further treatment.

**Figure:**
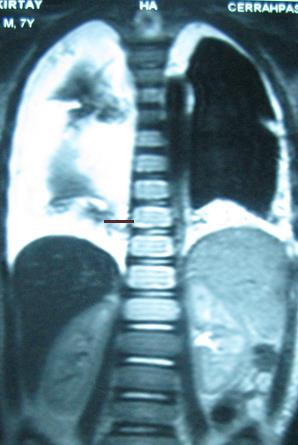
Figure 1: Bilateral pleural effusion and patchy osteolytic changes in thoracic vertebrae

Gorham Syndrome associated with chylothorax may be fatal especially with bilateral pleural effusion mortality rates are very high.[4] Hypoproteinemia, malnutrition, immunosuppression and lymphocytopenia may be the causes of death. There is no standard treatment modality for Gorham syndrome with chylothorax. Pleurectomy, pleurodesis, thoracic duct ligation, radiation therapy, somatostatin, steroid and interferon are suggested treatment modalities.[5,6] In our case, as others mentioned in the literature, steroid and somatostatin treatment were tried but failed. Many reports show succesfull results with α-interferon treatment. In 1997, Hagberg et al succeeded in treating the disease with α-interferon due to the antiangiogenic features of the drug and prevention of proliferation of vessels.[7] Kose et al in 2011 reported the use interferon alpha-2b for chylothorax. [3] We also used interferon alfa-2b with quite promising results. No complications were noted.

To conclude, Gorham syndrome with bilateral recurrent chylothorax is a tricky situation where treatment has to be tailored according to the response obtained. We have observed good outcome in the index case with interferon alfa-2b.

## Footnotes

**Source of Support:** Nil

**Conflict of Interest:** None declared

